# The bacterial consortia promote plant growth and secondary metabolite accumulation in *Astragalus mongholicus* under drought stress

**DOI:** 10.1186/s12870-022-03859-4

**Published:** 2022-10-07

**Authors:** Yixian Lin, Hui Zhang, Peirong Li, Juan Jin, Zhefei Li

**Affiliations:** State Key Laboratory of Crop Stress Biology in Arid Areas, Shaanxi Key Laboratory of Agricultural and Environmental Microbiology, College of Life Science, Northwest A&F University, Yangling, Shaanxi China

**Keywords:** Bacterial consortia, Secondary metabolite, Drought stress, Plant growth promoting bacteria

## Abstract

*Astragalus mongholicus* is a widely used Traditional Chinese Medicine. However, cultivated *A. mongholicus* is often threatened by water shortage at all growth stage, and the content of medicinal compounds of cultivated *A. mongholicus* is much lower than that of wild plants. To alleviate drought stress on *A. mongholicus* and improve the accumulation of medicinal components in roots of *A. mongholicus*, we combined different bacteria with plant growth promotion or abiotic stress resistance characteristics and evaluated the role of bacterial consortium in helping plants tolerate drought stress and improving medicinal component content in roots simultaneously. Through the determination of 429 bacterial strains, it was found that 97 isolates had phosphate solubilizing ability, 63 isolates could release potassium from potash feldspar, 123 isolates could produce IAA, 58 isolates could synthesize ACC deaminase, and 21 isolates could secret siderophore. Eight bacterial consortia were constructed with 25 bacterial isolates with more than three functions or strong growth promoting ability, and six out of eight bacterial consortia significantly improved the root dry weight. However, only consortium 6 could increase the root biomass, astragaloside IV and calycosin-7-glucoside content in roots simultaneously. Under drought challenge, the consortium 6 could still perform these functions. Compared with non-inoculated plants, the root dry weight of consortium inoculated-plants increased by 120.0% and 78.8% under mild and moderate drought stress, the total content of astragaloside IV increased by 183.83% and 164.97% under moderate and severe drought stress, calycosin-7-glucoside content increased by 86.60%, 148.56% and 111.45% under mild, moderate and severe drought stress, respectively. Meanwhile, consortium inoculation resulted in a decrease in MDA level, while soluble protein and proline content and SOD, POD and CAT activities increased. These findings provide novel insights about multiple bacterial combinations to improve drought stress responses and contribute to accumulate more medicinal compounds.

**Supplementary information:**

The online version contains supplementary material available at 10.1186/s12870-022-03859-4.

## Introduction

*Astragalus membranaceus* (Fisch.) Bge. var. *mongholicus* (Bge.) Hsiao (Shorthand for Astragalus) is one of important Traditional Chinese Medicine since the dried roots are abundant in pharmacological compounds, such as astragalosides (astragaloside I, astragaloside II, astragaloside III, and astragaloside IV), flavonoids (formononetin, calycosin-7-glucoside, calycosin, and ononin), and polysaccharides which can be used to regulate the immune system and have anti-inflammatory and anti-tumour properties [1–3]. Astragaloside IV and calycosin-7-glucoside have better therapeutic effects, thus the quality of Astragalus is evaluated by the content of these two compounds according to “Chinese pharmacopeia criterion”. Owing to its medicinal effects, Astragalus is the main raw material of more than 200 kind of Chinese patent drugs. Due to over exploitation, wild Astragalus resources are increasingly scarce and it was listed as “China Rare Endangered Plant Directory” [4]. At present, cultivated Astragalus are the main source of the market supply, however, the content of medicinal second metabolites in cultivated Astragalus is obviously lower than that of the wild plants. Meanwhile, the production areas of Astragalus are mainly distributed in the arid and semi-arid regions of northern China [5], water deficit environment exhibits deleterious effects to Astragalus growth. Therefore, there is great economic and social value to find an effective strategy to enhance plant biomass and the content of medicinal secondary metabolites of cultivated Astragalus in regions with lower precipitation.

Under water shortage conditions, crop regulates some physiological and biochemical process to adapt water deficit, such as regulating osmotic pressure by accumulating compatible solutes, producing phytohormones, synthesizing antioxidants, adjusting stomatal conductance, and decreasing transpiration rate and photosynthesis rate [6]. These physiological and biochemical changes disrupt the balance of primary and secondary metabolite synthesis, thus the secondary metabolite synthesis pathway of plants may be affected under drought and other abiotic stress [7]. For instance, drought stress induced an increase in production of sesquiterpene in *Salvia dolomitica* [8]. The production of polyamine derivatives and terpenoid blumenol of barley was found to be closely related with water deficit [9]. In addition to affecting secondary metabolite content, drought stress usually poses the detrimental effect on crop growth and development. Several methods have been used to increase the Astragalus biomass and medicinal secondary metabolite content including the use of agricultural plastic films, chemical fertilizer and imitation wild cultivation. Although plastic film mulching can keep soil moisture and increases crop yield, the increasing residuals of plastic debris and phthalate destroyed soil structure and affected agricultural environment [10]. Furthermore, overuse of chemical fertilizer has decreased the soil quality and soil microbial diversity, which in turn affects the crop quality and reduces crop yield [11, 12]. The imitation wild cultivation can improve quality of Astragalus, but this strategy has not been widely used because of the long planting time.

Increasingly studies showed that some beneficial microorganisms could increase medicinal plant yield and secondary metabolite content [13–15]. Plants recruit and feed specific microbes to colonize in the rhizosphere by secreting root exudates under drought stress [16–18]. In return, these rhizosphere microorganisms can promote plant growth under drought via multiple mechanisms, including production indole-3-acetic acid (IAA); dissolution insoluble phosphorus compounds and other mineral nutrients; fixation nitrogen; and secretion siderophores [19, 20]. Moreover, root associated microbes can assist plant to withstand drought stress through production of cytokinins, antioxidants and degradation of the ethylene precursor 1-aminocyclopropane-1-carboxylate [21]. For example, Chaín et al. [22] found that co-inoculation with *Pseudomonas* sp. M25 and *Pseudomonas* sp. N33 increased *E. grandis* growth and photosynthetic rate under drought. Besides plant growth promoting and drought resistance, plant-associated microorganisms stimulate the accumulation of medicinal secondary metabolites has also gained more attention. It is found that the contents of flavonoids, terpenoids, saponins and phenolic compounds in medicinal plants could be increased by inoculation endophytes [23–26]. Several studies have confirmed that some bacteria, such as *Bacillus pumilus*, *Streptomyces* sp., *Stenotrophomonas* sp., and *Pseudomonas fluorescens* were able to promote plant growth and elevate secondary metabolite production [27–29]. Although the mechanisms by which beneficial microbes promote medicinal plants accumulating secondary metabolites are not fully understood, the following ways are recognized by most people (i) endophytes can directly biosynthesize the secondary metabolites; (ii) endophytes can produce the precursor of secondary metabolites; (iii) some microbes involve in the transformation of secondary metabolites in medicinal plants; (iv) and microbe-plant interaction increases the content of secondary metabolites in plants [30]. Although a large number of studies have shown that single beneficial microorganism can promote plant growth, enhance plant tolerance to drought and increase the content of secondary metabolites in plant [31], few studies have focused on amelioration of abiotic stress and accumulation of secondary metabolites in plants with different functional bacteria at the same time.

In northwest China, Astragalus has special regional importance. But low annual precipitation limits plant growth, and the content of medicinal compounds in cultivated Astragalus is lower than that in wild plants. However, information is scarce regarding the role of beneficial bacteria in drought mitigation, plant growth promotion and medicinal secondary metabolite accumulation in Astragalus. The objectives of the this study were to (i) assess the effects of bacterial consortium on Astragalus growth; (ii) examine the mitigation effects of bacterial consortium on drought and accumulation of main medicinal components of Astragalus.

## Results

### Determination of bacterial isolates growth promoting characteristics

A total of 123 bacteria were isolated from rhizosphere and root of Astragalus, together with 306 bacterial strains previously screened from rhizosphere and root of Astragalus in other experiment, we obtained a bacterial bank containing 429 isolates. 16S rDNA sequence alignment showed that these isolates belonged to 61 genera, among which *Pseudomonas* (17.95%), *Microbacterium* (7.70%), *Rhizobium* (6.53%), *Bacillus* (6.29%), and *Brevibacterium* (6.06%) accounted for a high proportion (Fig. S1). Among these bacteria, 97 and 63 bacterial strains could dissolve inorganic phosphate and potassium in present study, respectively. However, only 7 and 5 bacteria had the D/d ratio greater than 2, suggesting that these strains had strong ability to dissolve inorganic phosphate or potassium. Quantitative determination indicated that the range of inorganic phosphate solubilization was between 92.56 and 624.4mg/L, and the concentration of soluble potassium in culture medium varied from 10.00 to 17.75mg/L (Fig. S2 and Table1). Twenty-one bacterial isolates were able to secrete siderophores based on CAS assay. Siderophores released by bacteria scavenged iron from Fe-CAS-hexadecyltrimethylammonium bromide complex, and formed yellow halo around bacterial colonies. The D/d ratio (Yellow halo diameter/ Colony diameter) of these isolates ranged from 1.08 to 2.33, and the strain HQB383 had the greatest D/d ratio (Table1). Among 429 bacterial isolates, 58 bacteria were able to grow on medium with ACC as a sole nitrogen source. The ACC deaminase activities of these 58 strains were in the range from 0.21 to 11.23 µmol α-ketobutyrate/h/mg protein (Fig. S2 and Table1), which suggested that these isolates had the potential to inhibit ethylene synthesis and thus enhance plant tolerance to abiotic stress. Moreover, IAA synthesis was a widespread feature for root-associated bacteria. Our results showed that 123 bacterial isolates had ability to produce IAA with a range from 0.86 to 5.33mg/L in kings medium.

### The effect of bacterial consortia on plant growth

**Fig. 1:**
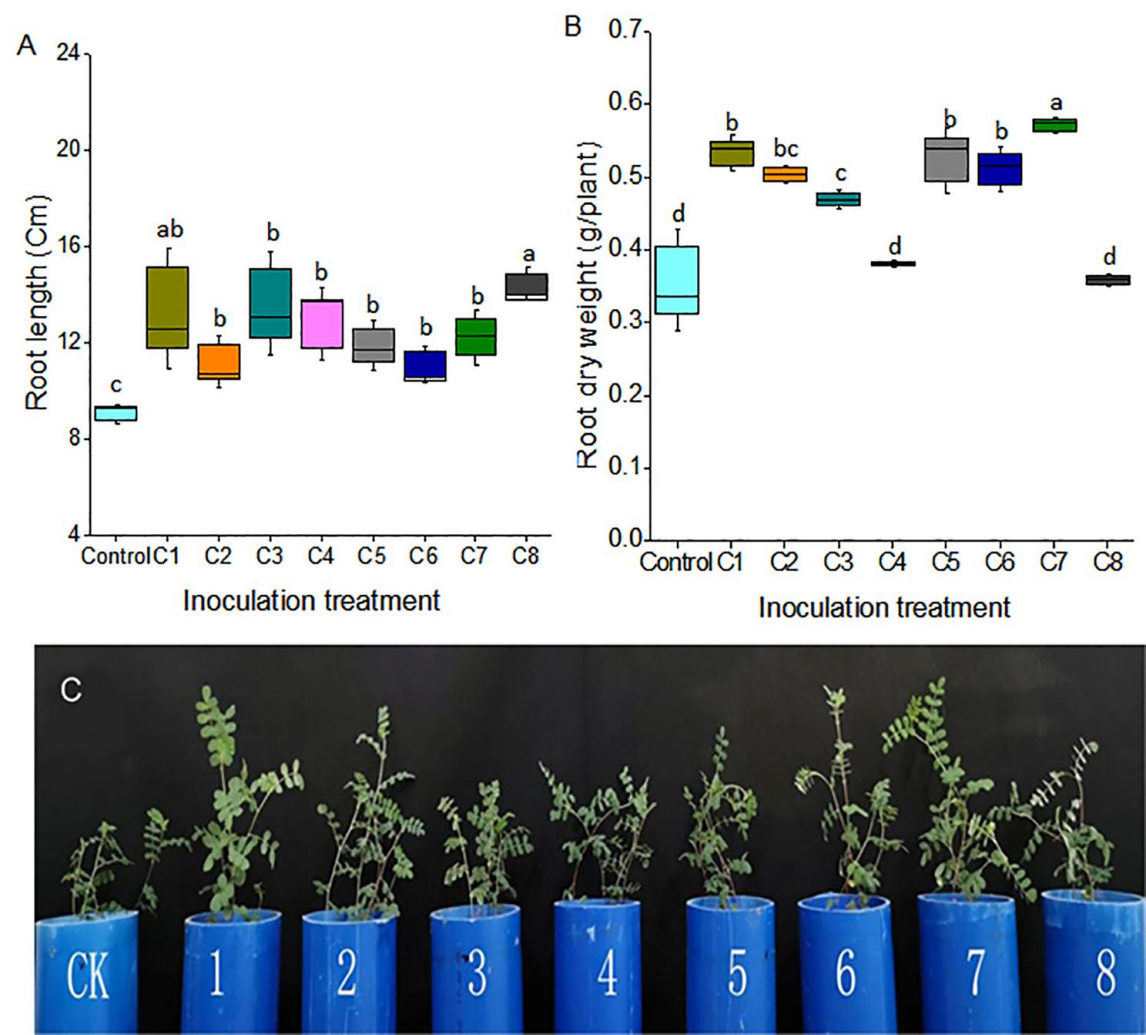
Effect of different bacterial consortia inoculation on plant growth. Root length (A) and root dry weight (B) of plants inoculated with different bacterial consortia and all plants grew for 5 months. (C) Phenotype of aboveground parts of plants grew for 2 months. Letters indicate significant differences (Root length, n = 9; Root dry weight, n = 4, P < 0.05). C1-C8, bacterial consortium 1- bacterial consortium 8

According to the growth promoting characteristics, the isolates with at least three functions, or with a relatively strong function were selected as candidate strains, and thus we obtained a total of 25 bacteria. Then we randomly constructed eight bacterial consortia (Abbreviated as C1-C8), including different numbers of bacteria. The bacterial strains contained in consortia 1-consortia 8 and their characteristics were shown in Table1 and Table S1. Compared with the control, bacterial inoculation treatments could promote the growth of Astragalus. All eight bacterial communities significantly increased the root length of Astragalus (Fig.1 and Table S2). Five out of eight bacterial consortia could increase the plant height and aboveground dry weight (Fig S3). Because the dry roots of Astragalus were used as medicinal raw materials, we paid more attention to the effect of bacterial consortia on the root biomass. The results showed that six consortia obviously increased the dry weight of roots, and consortium 7 could accumulate the maximum dry weight of the roots. Root dry biomass was increased by 47.22%, 44.44%, 41.67% and 58.33% (P < 0.01), respectively, in plants inoculated with consortium 1, consortium 5, consortium 6 and consortium 7 compared with non-inoculation plants (Fig.1B).

**Fig. 2:**
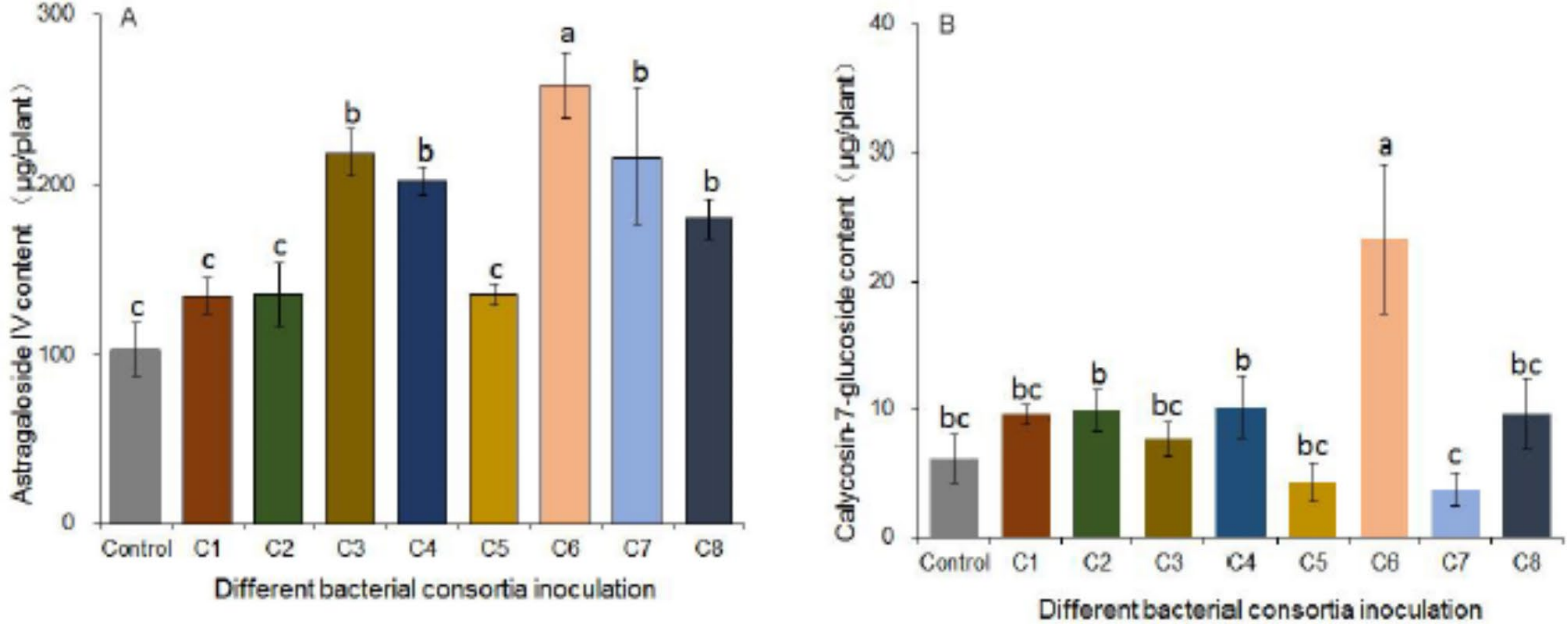
Effect of different bacterial consortia inoculation on the accumulation of medicinal metabolites in Astragalus. The total content of Astragaloside IV (A) and Calycosin-7-glucoside (B) in roots inoculated with different bacterial consortia, and all plants grew for 5 months. Letters indicate significant differences (n = 4, P < 0.05). C1-C8, bacterial consortium 1- bacterial consortium 8

In addition, we also analyzed the effect of different inoculation treatments on the accumulation of medicinal secondary metabolites in roots after the plants grew for five months. Most inoculation treatments could improve the content of medicinal constituents, but the accumulation of astragaloside IV and Calycosin-7-glucoside in roots inoculated with the same bacterial consortia was inconsistent. Consortium 3, consortium 4, consortium 6, consortium 7 and consortium 8 could significantly increase the astragaloside IV content, while only consortium 6 could promote the accumulation of calycosin-7-glucoside. Compare with the control, the total content of astragaloside IV and calycosin-7-glucoside in the plant roots inoculated with consortium 6 increased by 150.16% and 277.22%, respectively (Fig.2).

Consortium 6 was composed with 15 isolates, and these bacteria belong to eight genera based on the sequencing results of the 16S rDNA gene (Table S1 and Fig. S4). The isolate HQB9, HQB56, HQB13, HQB383, HQB90, and HQB216 belong to *Bacillus*, *Acinetobacter*, *Leclercia*, *Sphingomonas*, *Microbacterium*, and *Brevibacterium*, respectively (GenBank accession number: OM903083, OM908937, OM903874, OM903864, OM903873, OM909062), the isolates HQB346 and HQB302 belong to *Rhodococcus* genus (Accession number: OM909072 and OM903864), while the isolates HQB 289, HQB19, HQB306, HQB290, HQB84, and HQB286 belong to *Pseudomonas* genus (Accession number: OM908924, OM908925, OM90892, OM908926, OM908922, and OM908923).

**Table 1:** Characteristics of the bacterial strains used to construct the consortia

Isolates	Genus	P content(mg/L)/ D/d	K content(mg/L)/ D/d	IAA content (mg/L)	ACC deaminase activity (µmol/mg•h)	Siderophore D/d
HQB9	*Bacillus* sp.	624.40 ± 76.07 / 1.43	14.92 ± 0.29 / 1.50	1.89 ± 0.03	2.87 ± 0.15	1.27
HQB56	*Acinetobacter* sp.	568.58 ± 55.53 / 1.33	-	-	2.56 ± 0.41	1.33
HQB32	*Variovorax* sp.	553.11 ± 21.58 / 1.50	-	-	4.71 ± 0.86	1.22
HQB13	*Leclercia* sp.	345.66 ± 17.49 / 2.33	11.58 ± 0.14 / 1.40	1.50 ± 0.07	-	1.4
HQB383	*Sphingomonas* sp.	232.50 ± 59.24 / 2.00	-	2.22 ± 0.12	-	2.33
HQB185	*Bosea* sp.	164.99 ± 40.09 / 1.50	-	1.65 ± 0.07	-	-
HQB41	*Bacillus* sp.	149.14 ± 45.32 / 1.20	10.00 ± 0.50 / 1.22	1.31 ± 0.06	0.80 ± 0.15	1.7
HQB125	*Rhizobium* sp.	140.84 ± 35.74 / 1.33	-	4.79 ± 0.10	-	-
HQB269	*Pantoea* sp.	136.32 ± 4.50 / 1.14	-	2.45 ± 0.09	-	1.27
HQB302	*Rhodococcus* sp.	133.68 ± 28.65 / 1.44	11.00 ± 1.00 / 1.25	1.42 ± 0.04	8.42 ± 1.27	-
HQB289	*Pseudomonas* sp.	131.42 ± 16.08 / 1.88	-	1.62 ± 0.07	0.36 ± 0.01	1.27
HQB17	*Bacillus* sp.	-	12.83 ± 0.52 / 2.00	0.86 ± 0.03	2.11 ± 0.73	1.11
HQB179	*Paenarthrobacter* sp.	121.23 ± 12.28 / 1.22	17.75 ± 0.43 / 1.71	1.43 ± 0.13	9.00 ± 0.22	1.14
HQB19	*Pseudomonas* sp.	116.33 ± 15.06 / 2.83	-	1.82 ± 0.07	-	1.43
HQB306	*Pseudomonas* sp.	112.93 ± 5.56 / 1.50	-	5.17 ± 0.11	-	1.13
HQB90	*Microbacterium* sp.	-	-	2.82 ± 0.07	8.70 ± 0.44	1.08
HQB33	*Variovorax* sp.	106.90 ± 9.96 / 1.80	-	-	11.23 ± 1.19	1.21
HQB6	*Achromobacter* sp.	105.01 ± 7.49 / 1.08	-	0.94 ± 0.03	4.08 ± 0.39	1.2
HQB2	*Brevibacterium* sp.	99.73 ± 16.37 / 2.17	13.50 ± 0.90 / 1.33	-	-	1.13
HQB290	*Pseudomonas* sp.	98.60 ± 12.59 / 1.07	-	3.23 ± 0.16	0.21 ± 0.02	1.24
HQB216	*Brevibacterium* sp.	-	12.17 ± 0.95 / 1.71	2.50 ± 0.06	2.39 ± 0.25	1.11
HQB97	*Pseudomonas* sp.	97.47 ± 28.71 / 1.50	11.67 ± 0.38 / 1.40	1.26 ± 0.06	-	1.13
HQB84	*Pseudomonas* sp.	96.34 ± 11.71 / 1.46	11.92 ± 0.29 / 1.29	2.43 ± 0.10	1.13 ± 0.09	1.25
HQB286	*Pseudomonas* sp.	94.07 ± 4.34 / 1.40	-	-	0.58 ± 0.04	1.41
HQB346	*Rhodococcus* sp.	92.56 ± 28.29 / 2.33	-	5.53 ± 0.13	-	-

Note: “-“ in the table means that the strain does not have this characteristic

### Plant tolerance to drought stress under bacterial consortium treatment

**Fig. 3:**
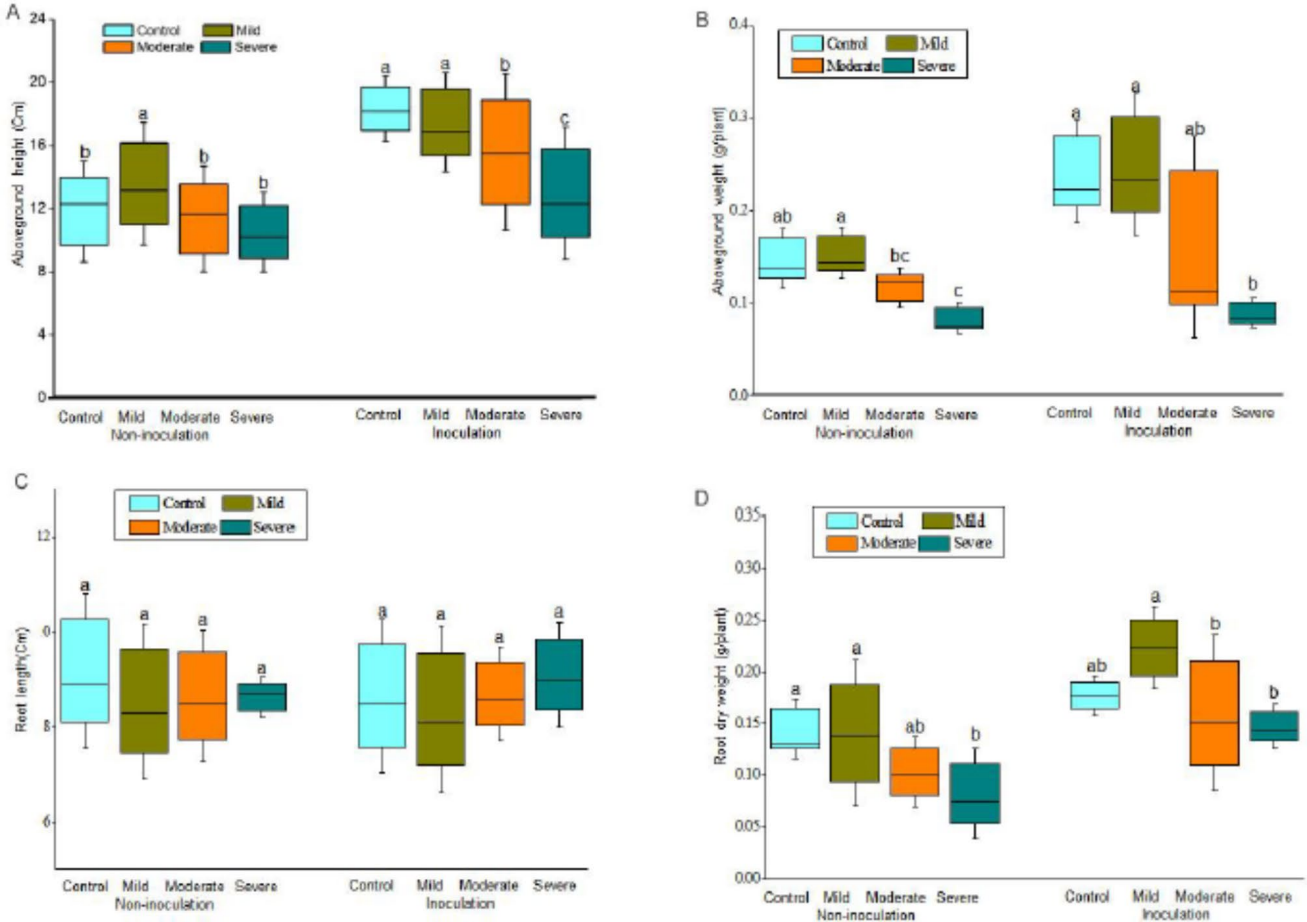
Effect of drought stress and consortium 6 on the plant growth. Plants grew in soil with different moisture levels and were not inoculated or inoculated with bacterial consortium 6. Aboveground height (A), aboveground dry weight (B), root length (C) and root dry weight (D) were determined after the plants grew for 30 days under drought stress. The letters indicated significant difference according to Tukey test, P ≤ 0.05

Since the bacterial consortium 6 could promote the root biomass and medicinal constituent accumulation in plants simultaneously, we validated whether this bacterial consortium could perform the same function under drought stress. As shown in Fig.3, whether bacterial inoculation or not, the root dry weight, aboveground height and aboveground dry weight showed a decrease trend with the reduction of soil water content. However, the bacterial consortium inoculation significantly increased plant aboveground height, root diameter and root dry weight at all conditions (Fig S5 and Table2). Compared with control, the root dry weight of inoculation plants increased by120.0% and 78.8% under mild and severe drought stress, respectively. Although bacterial inoculation had no effect on root length, it increased the root diameter which leaded to a significant accumulation of root dry weight (Fig S5).

## Effects of drought stress and bacterial consortium on accumulation of medicinal constituents

**Fig. 4:**
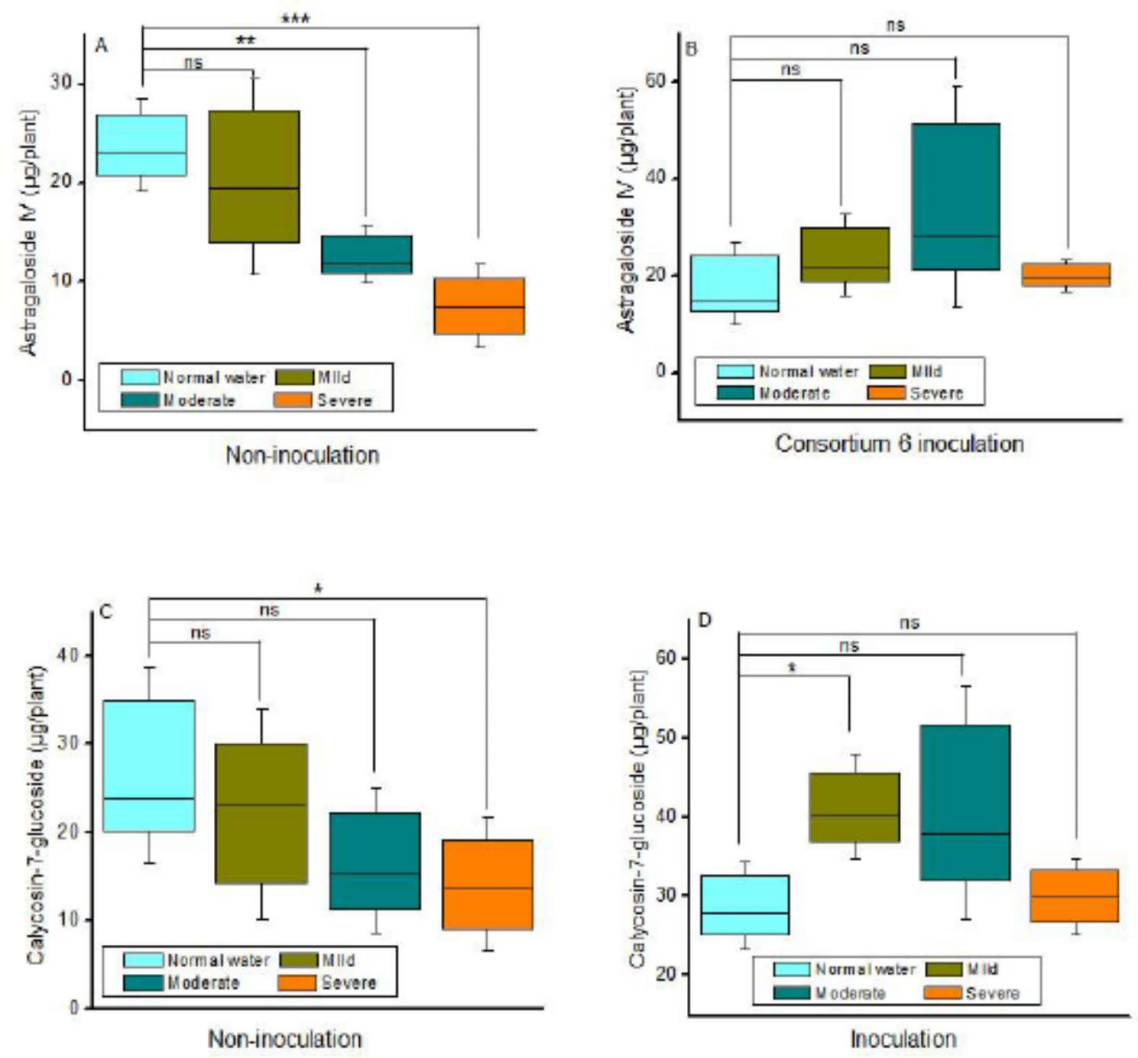
Effect of drought stress and consortium 6 on the calycosin-7-glucoside and astragaloside IV content. Plants grew in soil for two months and subjected to different drought levels for 30 days. The total content of astragaloside IV (A, B) and calycosin-7-glucoside (C, D) in roots of Astragalus was determined, each treatment with four replications. ‘ns’ indicated no significant difference. The asterisks indicated significant difference according to Student’s t test, ‘*’: P ≤ 0.05; ‘**’: P ≤ 0.01; ‘**’: P ≤ 0.001

Two months old plants were subjected to mild, moderate and severe drought stress for 30 consecutive days, and the total content of astragaloside IV and calycosin-7-glucoside in the roots were determined. Without bacterial inoculation, moderate and severe drought treatment could decrease the total content of astragaloside IV and calycosin-7-glucoside in Astragalus, but there was no significant difference between mild drought and normal watering plants. However, the consortium 6 significantly increased the content of calycosin-7-glucoside in Astragalus under mild drought stress, and the calycosin-7-glucoside content was 43.42% more than that of control plants. Although bacterial consortium 6 did not significantly increase the content of medicinal constituents (except for calycosin-7-glucoside under mild drought), bacterial inoculation prevented the decrease of medicinal constituents in Astragalus under drought stress. Meanwhile, the calycosin-7-glucoside and astragaloside IV content of inoculated plants was obviously higher than that of non-inoculated plants under moderate and severe drought stress (Fig.4 and Fig S6). Compared with normal watering plants, the calycosin-7-glucoside and astragaloside IV content of non-inoculated Astragalus under moderate stress reduced by 38.99% and 46.60%, while these two metabolites of bacterial consortium inoculated-Astragalus increased by 45.07% and 97.50%, respectively.

## The content of MDA, proline and soluble proteins response to bacterial consortium inoculation

**Fig. 5:**
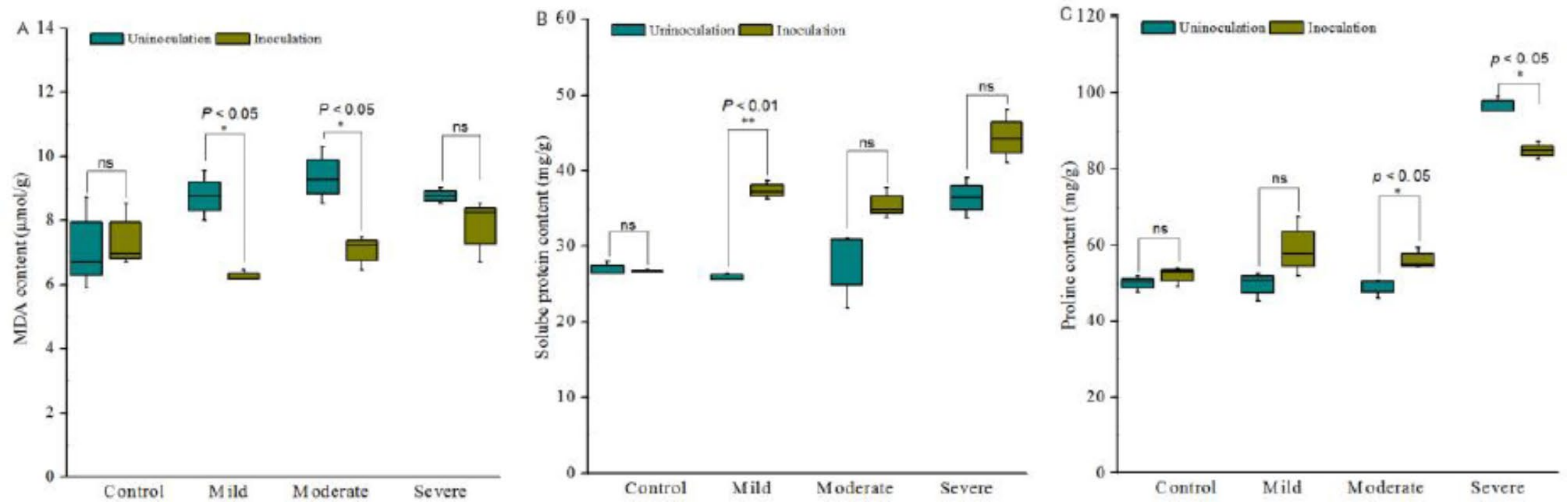
The MDA, proline and soluble protein content of plants response to drought and bacterial inoculation. Plants grew in soil for two months and subjected to different drought levels for 30 days, the MDA, soluble proteins and proline content of un-inoculated Astragalus (dark green) and consortium 6 inoculated Astragalus (earth yellow) under the different level of drought were determined. Each treatment had four replications. ‘ns’ indicated no significant difference. The asterisks indicated significant difference according to Student’s t test, ‘*’: P ≤ 0.05; ‘**’: P ≤ 0.01

Drought generated reactive oxygen species (ROS) that caused lipid peroxidation and increased the production of malondialdehyde (MDA). Thus, MDA content is an important indicator of lipid damage under the drought condition [32]. In this study, MDA level in plants increased from mild drought stress to severe drought stress. As response to bacterial consortium 6 inoculation, a significant decrease in MDA level was detected in Astragalus under mild and moderate drought stress (*p* < 0.05). In these two irrigation strategies, the content of MDA in inoculated plant was 28.39% and 24.76% less than non-inoculated plant, respectively (Fig.5A).

The inoculation of consortium 6 did not affect the content of proline and soluble proteins of Astragalus at well watering conditions. Under mild or moderate drought condition, the proline and soluble content increased dramatically. Except the proline content in bacterial consortium 6 treated plants was less than that of un-inoculated plants under severe drought stress, the soluble protein and proline level of the inoculated plants was significantly higher than that of the un-inoculated plants at the mild drought and moderate drought stress, respectively (Fig.5B C).

**Table 2:** Effect of bacterial consortium 6 on plant growth

Treatment	Root length		Plant height		Root fresh weight		Root dry weight		Root diameter		Aboveground fresh weight		Aboveground dry weight	
	t	*P*	t	*P*	t	*P*	t	*P*	t	*P*	t	*P*	t	*P*
**Normal watering**														
Consortium 6 VS control	**3.68**	0.003**	**7.64**	< 0.001***	**6.08**	0.004**	**5.17**	0.007**	**2.63**	0.03*	**3.44**	0.03*	**2.93**	0.04*
**Mild drought**														
Consortium 6 VS control	0.63	0.54	**5.23**	< 0.001***	**4.89**	0.003**	**3.18**	0.02*	**4.54**	< 0.001***	1.39	0.25	**3.55**	0.01*
**Moderate drought**														
Consortium 6 VS control	0.93	0.36	**5.73**	< 0.001***	2.37	0.056	**2.82**	0.05*	**4.59**	< 0.001***	**3.15**	0.02*	1.48	0.23
**Severe drought**														
Consortium 6 VS control	1.02	0.31	**3.68**	0.001**	2.07	0.084	**4.04**	0.007**	**2.74**	0.009**	1.55	0.17	0.69	0.52

Note: Plant grew in greenhouse for 2 months, then subjected to drought stress for 30 days. Bold values in columns indicated significantly different at *P* ≤ 0.05 according to t-Test. ‘*’: *P* ≤ 0.05; ‘**’: P ≤ 0.01; ‘***’: *P* ≤ 0.001

## The antioxidant enzyme activities response to bacterial consortium inoculation

**Fig. 6:**
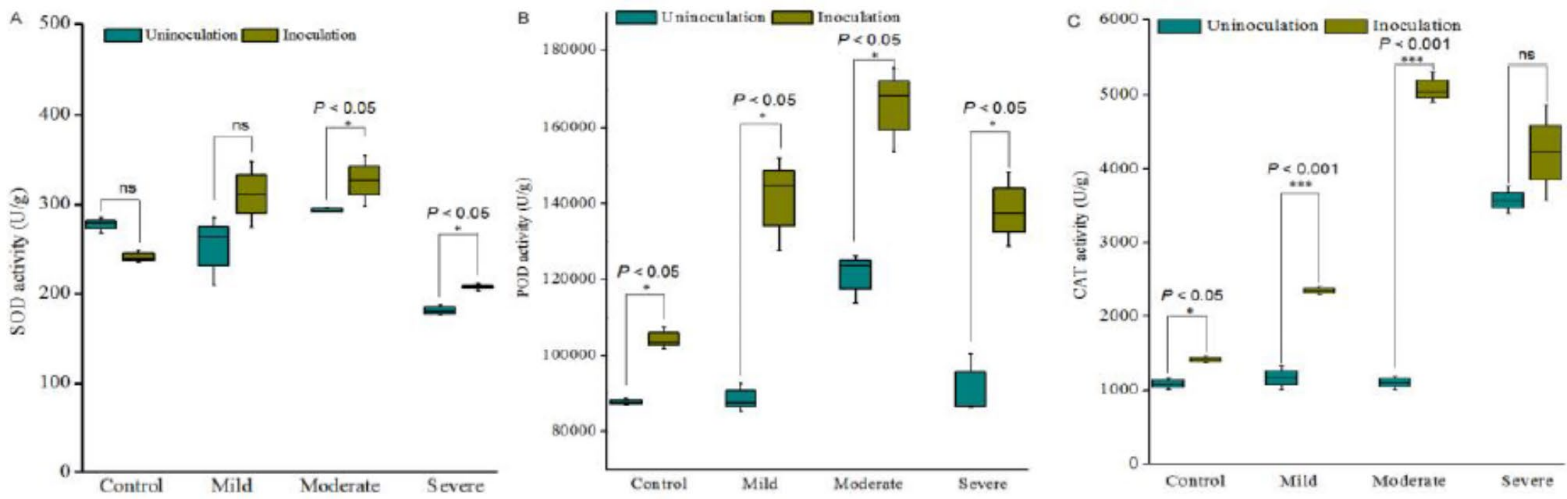
The antioxidant enzyme activities of plants response to different drought level stress and bacterial inoculation. Plants grew in soil for two months and subjected to different drought levels for 30 days, the activities of SOD, POD and CAT of non-inoculated Astragalus (dark green) and consortium 6 inoculated Astragalus (earth yellow) under the different level of drought were determined. Each treatment had four replications. ‘ns’ indicated no significant difference. The asterisks indicated significant difference according to Student’s t test, ‘*’: P ≤ 0.05; ‘**’: P ≤ 0.01; ‘***’: P ≤ 0.001

As shown in Fig.6, the activities of SOD and POD increased and then decreased from well-watered to severe drought stress, which reached the maximum under moderate drought stress. The SOD and POD activities of the inoculated Astragalus were much higher than that of the non-inoculated Astragalus (Fig.6), and the activities of SOD and POD increased by 11.24% and 36.75% under moderate drought stress, respectively (p < 0.05). However, CAT activities of un-inoculated plants reached the maximum under severe drought stress, the enzyme activities of inoculated plants in mild and moderate drought were significantly higher than that of non-inoculated plants (P < 0.001). The CAT activities of the bacterial inoculated Astragalus also reached the maximum under moderate drought condition, and the CAT activities of inoculated plant were 33.60% more than non-inoculated plants (Fig.6).

## Discussion

### The role of growth-promoting bacteria in plant biomass accumulation

It is well known that the plant rhizosphere and root provides nutrients and niches for a huge and diverse microbial survival. Among the root associated microbes, some beneficial bacteria, known as plant growth-promoting bacteria (PGPB), can promote plant growth by direct and indirect mechanisms. Direct promotion of plant growth involves increasing the bioavailability of nutrients such as nitrogen, phosphorous, potassium and iron as well as enhancing hormone biosynthesis [33]. PGPB indirectly promote plant growth by improving plant tolerance to abiotic stress [34] or inhibiting the growth of pathogens [35]. Most researchers have demonstrated that microbial inoculation can promote the growth of various plants. Considering the complex interaction among microbes, the growth-promoting effect of multi-bacterial inoculation may be different from that of individual bacterium inoculation. Thus, the bacterial strains with the properties of dissolving inorganic phosphorus, releasing potassium, producing siderophores and synthesizing IAA were screened from Astragalus rhizosphere and root in present study. We combined bacteria with multiple functions or strong growth-promoting functions and conducted experiments in a greenhouse. The results of pot-based experiments indicated that six consortia significantly improved the root dry biomass. Most bacteria in these consortia belonged to *Bacillus*, *Pseudomonas*, and *Sphingomonas* genus, and these groups of microbia have been shown to have growth-promoting effects in a variety of plants [36, 37]. Before the experiments, we did not know which isolates can play a role in promoting plant growth, alleviating drought stress to *A. mongholicus* and inducing *A. mongholicus* to accumulate astragaloside IV and calycosin-7-glucoside, so we only inoculated *A. mongholicus* with bacterial consortia without individual bacterium for comparation. Since consortium 6 can perform the functions we desired, we will compare the effects between consortium and individual bacterium, and investigate the functional superposition and synergism among bacterial strains in the future. Although all the selected strains had growth-promoting properties, some consortia did not increase plant biomass, this may be due to competition between certain strains in the consortia.

## The role of PGPB in secondary metabolite synthesis

It was reported that rhizosphere or endophytic microorganisms could promote the accumulation of secondary metabolites in medicinal plants [38]. The results of our study showed that there was no significant difference in the total content of astragaloside IV and calycosin-7-glucoside between control and Astragalus inoculated with bacteria under normal watering conditions. However, bacterial consortium 6 could promote the accumulation of these two secondary metabolites in plants under drought stress. This is consistent with other studies in which *Bacillus pumilus* could markedly increase the expression of relative genes and induced the production of glycyrrhizic acid [39].

It is generally believed that drought stress has an adverse effects on plant growth. However, it has been noticed that some secondary metabolites can improve drought tolerance of medicinal plants. For instance, the carnosic acid, a diterpen compound, was responsible for mitigating drought-induced damage in rosemary [40]. To improve drought resistance, *Hypericum brasiliense* accumulated high level flavonoids such as rutin and quercetin [41]. Studies have shown that secondary metabolites enhance drought resistance in plants in a variety of mechanisms. Flavonoids and polyphenols promote plant to scavenge ROS induced by drought stress [42]. In addition, secondary metabolites have been found to enhance the TCA cycle and activate the proline biosynthesis pathway, which enhances the adaptability of plants to abiotic stress [43]. In present study, we found that the concentration of astragaloside IV and calycosin-7-glucoside in roots of bacteria-inoculated plants was significantly higher than that of un-inoculated plants, and bacterial consortium 6 could also improve the tolerance of Astragalus to drought stress. Therefore, whether these secondary metabolites are related to drought resistance in plants, and how astragaloside IV and calycosin-7-glucoside alleviates drought damage in Astragalus needs to be further investigated in future work.

## The role of PGPBs in drought tolerance

In nature, water deficit or drought is considered as the most severe abiotic factors limiting various crop growth and yield. Although many plants survive in water deficit environment through morphological change, physiological acclimation, and intracellular molecular and biochemical process adjustment [44], they also pay the price of decreased leaf area, slow growth and reduced biomass. More and more studies have shown that plant related microbes can alleviate the adverse effects of biotic and abiotic factors on plants, which provides an alternative method to increase crop yield in arid areas. Several studies have indicated that the beneficial microbes relieve the negative impact of drought stress on crop growth, at least in part due to the PGPBs can produce IAA, ACC- deaminase and exopolysaccharide [45]. In our study, the bacterial consortium 6 consists of 15 PGPBs, of which 9 and 13 bacterial strains can produce ACC-deaminase and IAA, respectively. Moreover, the ACC-deaminase activities of strain HQB90 (*Microbacterium* sp., 8.70 µmol/mg•h) and HQB302 (*Rhodococcus* sp., 8.42 µmol/mg•h) were much higher than that of other bacteria, while the strain HQB306 (*Pseudomonas* sp., 5.17mg/L) and HQB346 (*Rhodococcus* sp., 5.53mg/L) produces much more IAA than other strains. ACC, the precursor of ethylene biosynthesis, generated by plants under abiotic stress is consumed by ACC-deaminase produce bacteria, resulting in the decrease of ethylene level in plants [46, 47]. Thus the bacterial consortium can enhance the absorption of nutrients, consequently promoting plant growth under drought stress. Besides ACC-deaminase, some evidences have shown that IAA can cross-talk with other phytohormones to regulate plant development, and then modulate the plant adaption to drought stress [48]. This may be one of the reasons why the bacterial consortium 6 can significant increase the plant height, root diameter and root dry weight of Astragalus under drought stress.

## Plant physiological and biochemical responses to PGPBs under drought stress

In plant cells, metabolic imbalance is triggered by biotic and abiotic stress leading to oxidative stress, by which produce ROS and attack cell membranes, lipoproteins and other lipid-containing structures causing lipid peroxidation. As the final product of lipid peroxidation, MDA content indicates the degree of oxidative damage which determines the severity of stress faced by plants [49]. In our study, the MDA content of bacterial consortium inoculated plants much lesser than that of un-inoculated plants under mild and moderate drought stress. Previous study has also shown that less MDA accumulation in *CkWRKY33*-overexpressing transgenic plants can enhance the survival rate of plants following a drought stress treatment [50].

Plants scavenge ROS via biosynthesizing antioxidant enzymes such as SOD, POD and CAT to reduce oxidative stress and cellular damage. Compared with drought-sensitive genotypes, several drought-tolerant plants accumulate higher levels of SOD, POD and CAT under drought stress [51]. Plants produce superoxide from photosynthetic and respiratory electron leakage under drought stress. Antioxidant enzymes are one of the important defense mechanisms of plants tolerance to abiotic stress. The SOD can dismutate superoxide into O_2_ and H_2_O_2_, then the POD and CAT scavenge H_2_O_2_ and decrease the ultimately oxidative stress. Researchers have also found a variety of bacteria and fungi that can improve plant tolerance to drought by improving activities of antioxidant enzymes in plants [52]. However, most studies use an individual microorganism to promote plant growth under drought conditions. Our study showed that a consortium composed of 15 plant growth-promoting bacteria could increase the dry biomass of Astragalus root, and the activities of antioxidant enzymes in plants was greatly increased under the condition of water deficit. These findings agree with the results of Shafi and Sarker who observed an increase in enzyme activities of SOD and CAT conferring salt or drought stress of plants [53, 54].

Besides antioxidant enzyme activities, our study showed that bacterial consortium inoculated Astragalus had a higher soluble protein and proline content under drought conditions. It is evident that the accumulation of osmotic regulatory substances, such as proline and soluble protein, play an important role in adapting osmotic stress and detoxification of ROS in plants. Environmental stress induces proline and soluble protein biosynthesis in plants to maintain cell turgor, balance the infiltration of protoplasm, and enable various physiological processes [55]. Numerous studies have shown that exogenous application of proline improves the adaption of plants to oxidative stress which induced by abiotic environmental stress [56]. Such adaption may be mediated by proline protecting membranes and proteins from damage by reactive oxygen species. Thus, our findings showed that the bacterial consortium inoculation might reduce the damage of plants by accumulating proline and soluble protein, decreasing the content of ethylene and improving the activity of antioxidant enzymes in Astragalus, and then enhance plant tolerance and adaptation to drought stress.

## Conclusion

In natural and agricultural ecosystem, plant growth and environmental adaptation is closely related to rhizosphere microorganisms. Therefore, we combined different PGPR with phosphate solubilizing, potassium solubilizing, ACC deaminase producing, siderophore and IAA biosynthesis traits. Under drought condition, the bacterial consortium 6 could significant promote plant growth and astragaloside IV and calycosin-7-glucoside accumulation in roots of *A. mongholicus*. Further studies showed that the consortium 6 could also increase antioxidant enzyme activity, synthesize proline and soluble protein to alleviate the adverse effects of drought on Astragalus. This results suggest that microorganisms with different functions can be combined into consortium to improve crop yield and quality in sustainable agriculture.

## Methods and materials

### Sampling collection

The plant and soil samples were collected from Astragalus plant fields in Tanchang county (N34°16.891’; E104°09.855’), Gansu province, China on May, 2019. Soil total nitrogen, total phosphorus, organic matter and pH were 1.72g/kg, 1.21g/kg, 24.3g/kg and 8.16, respectively. Astragalus were uprooted randomly with a spade after removing plant debris. The samples were immediately transported to the laboratory. Then the plants were shaken vigorously to remove loose soil bound to the roots, the 1–2mm thick rhizosphere soil surrounding the roots was brushed into sterile plastic bags and stored at -80°C until bacterial isolation.

## Isolation of root endophytes and rhizosphere bacteria

The roots were disinfected by wiping with 6% sodium hypochlorite, followed by washing roots with sterile water for 6 times and then grounding into homogenate in the sterilization mortar. The isolation of the bacteria was done as follows: 1g rhizosphere soil or 1 mL root homogenate was added in 9 mL sterile distilled water and incubated on rotary shaker at 180rpm for 15min. The soil suspension was serially diluted to 10^− 7^, then 10 µL 10^− 5^, 10^− 6^ and 10^− 7^ dilution of soil or root tissue suspension was plated on LB (Tryptone: 10g, Yeast extract: 5g, NaCl: 5g, Agar: 15g, Water: 1000 ml) or TSA agar (Casein peptone: 15g, soya peptone: 5g, NaCl: 5g, Agar: 15g, Water: 1000 ml) and incubated for 3–7 days at 28℃. Single colonies were picked up and streaked on sterile LB agar plates to get pure culture. The genomic DNA of all isolates was extracted according to the method of Wilson & Carson [57]. The 16S rDNA region was amplified using forward primer (5-AGAGTTTGATCCTGGCTC) and reverse primer (5-CGGCTACCTTGTTACGACTT). The amplified fragments were detected by agarose gel electrophoresis, and then the correct amplification products were sequenced in Sangon Biotech (Shanghai) Co., Ltd, China. The obtained DNA sequences were aligned with sequences present in the gene database bank by BLASTN program.

## Screening for plant-growth-promoting traits

### Production of IAA

Bacterial isolates were cultured in 50 mL Kings medium supplemented with tryptophan (50mg/L) and incubated at 28°C for 72h on a rotary shaker. Bacterial suspension was centrifuged at 10,000*g* for 10min. One milliliter supernatant was transferred into EP tube containing equal volume of Salkowski reagent. After 15min, the pink color of the reaction liquid indicated that the corresponding isolate was positive bacterium for producing IAA. Then, 5 mL of each positive bacterial supernatant was transferred to a separating funnel, equal volume of ethyl acetate was added and extracted for 1h. The solvent in the extraction supernatant evaporated and the residue was fully dissolved with 1 mL chromatographic methanol. The content of IAA was determined by HPLC after the samples were filtered with 0.45 µL Millipore according to the method of Sheikhian [58].

### ACC deaminase activity assay

For ACC deaminase activity of bacteria determination, the bacteria were inoculated in 20 mL LB liquid medium and grew to log phase. Then the bacterial solutions were inoculated in ADF medium supplement with 1-aminocyclopropane-1-carboxylate (ACC) as sole nitrogen source. Seven milliliter suspension from tube in which bacterium could grow by metabolizing ACC was centrifuged at 8,000*g* for 5min and the sediment was washed with 5 mL Tris-HCl (0.1mol/L, pH = 7.5) twice. 600 µL Tris-HCl (0.1mol/L, pH = 8.5) and 30 µL methanol was added to the precipitate and ultrasonic extraction for 30s to obtain the crude enzyme solution. Then 20 µL ACC (0.5mol/L) was added in 100 µL crude enzyme solution and reacted for 15min. The reaction solution was centrifuged for 5min at 14,000*g*, then the content of α-ketobutyrate in supernatant was determined by measuring the absorbance of the sample at 540nm and calculating according to standard curve of α-ketobutyrate ranging from 0.1 to 1.0 mmol/L. The ACC deaminase activity was defined as the amount of α-ketobutyrate generated per mg of protein per hour.

### Solubilization of phosphate and potassium

Five microliter suspension of each bacteria was spotted on PVK or Aleksandrov agar plates, respectively. After 5 days of plate incubation, the bacterial colonies with clear zone were phosphate/potassium-soluble bacteria and D (Diameter of zone of clearance) / d (Diameter of colony) represents the capacity of bacteria to dissolve phosphate or potassium. Then, the phosphate/potassium-soluble strains were inoculated into the corresponding liquid medium and incubated for 5 days at 28°C, 180rpm. The available phosphorus concentration in the suspensions were evaluated using the molybdovanadate method [59]. The available potassium concentration in the suspensions were determined using atomic absorption spectrometry [60].

### Siderophore production

Siderophore production was detected using Chrome Azurol-S (CAS) medium according to the method described by Louden [61]. Ten microliter bacterial suspension was spotted on the CAS agar plates and incubated at 28°C for 5 days. The ratio of the diameter of yellow-orange halo around bacteria (D) to the diameter of bacterial colony was considered as an indicator for siderophore production.

## Pot experiment

Eight milliliter logarithmic bacterial suspension was added into tissue culture vessel containing 80g of sterilized peat and fermented for 7 days. In this way, a total of 25 bacterial agents were obtained. According to the composition of 8 designed bacterial consortia, 1g of corresponding bacterial agents was taken and mixed evenly to produce synthetic bacterial communities (Table S2). The sterile growth substrate (soil: vermiculite = 10:3, w/v) was inoculated with different bacterial communities at 2.5%, and the control growth medium added equal quality peat, three replicates were used for each treatment. Each plastic containers filled with 1kg growth medium. Three Astragalus seedlings were planted in each plastic container and grew in glasshouse at a 16/8h photoperiod with a temperature of 23/18°C (day/night). Five-months-old plants were harvested, the plant height, root length, plant fresh weight, plant dry weight and content of medicinal constituents were determined.

## Drought stress experiment

The growth substrate was inoculated with a bacterial community that could significantly promote plant growth and medicinal secondary metabolite accumulation. Astragalus seedlings grew for two months, then the plants were subjected to drought stress: mild drought, the soil moisture content is about 25%; moderate drought, the soil moisture content is about 15%; severe drought, the soil moisture content is about 10%; the well-watered plants used as control, each treatments contained four pots and five germinated seedlings were sown in a pot. Other culture conditions were described as above. The plant height, root length, plant fresh weight, plant dry weight and content of medicinal constituents were measured after one month growth.

## Measurement of medicinal constituent content

Five milligram standard substance of astragaloside or flavonoid was dissolved in methanol to a final volume of 10 mL. The solutions were diluted with methanol to prepare 5, 10, 25, 50, 100, and 200µg mL^− 1^ solutions. The content of saponin and flavonoid in roots was determined according to the method of Xu [62]. Briefly, 0.5g dry roots were ground to powder and transferred in a 25 mL centrifuge tube with 10 mL methanol. The mixtures were sonicated for 2h at 40Hz, 100W (KS-250DE ultrasonic cleaner). The extracting solutions were centrifuged twice at 12,000g for 10min and the supernatants were filtered with 0.22μm microporous membrane. Saponin and flavonoid content of all samples was determined with LC-MS system (QTRAP 5500, AB Sciex, MA, USA) equipped with a triple quadrupole mass spectrometer detector (QQQ), and an electrospray ion source. The operating parameters of liquid chromatography and mass spectrometry referred to the method of Jiao [63].

## Evaluation of plant physiological and biochemical response to consortium inoculation

### MDA content analysis

Plant samples (0.1g) were homogenized in 1 mL pre-cooled 10% trichloroacetic acid solution (w/v). The homogenate was centrifuged at 8,000g for 10min and 0.1 mL supernatant was added 0.3 mL 0.5% thiobarbituric acid (w/v). The mixture was incubated in 95°C water for 30min, then the reaction solution was cooled and centrifuged at 10,000g for 10min. The absorbance of the supernatant was measured at 450, 532, and 600nm, and the MDA content was calculated as: MDA content (µmol g^− 1^ FW) = [6.452 × (D_532_ − D_600_) − 0.56D_450_] × V × (W × 1000) ^−1^, where V is the volume of the extract.

## Proline content analysis

The samples (0.1g) were homogenized in 1 mL 3% aqueous sulfosalicylic acid. The homogenate was incubated in boiling water for 10min. The solution was centrifuged at 10,000g for 10min. The supernatant (0.25 mL) was added in 0.25 ml glacial acetic acid and 0.25 ml acidic ninhydrin, and the mixture were kept in boiling water bath for 10min. The reaction solution was added in 0.5 ml toluene. The absorbance of the solution was determined at 520nm and the proline content was calculated using standard curve.

## Soluble protein analysis

Plant samples (0.1g) placed in 1 mL distilled water and homogenized in ice bath, the mixture was centrifuged at 10,000g for 10min. One milliliter supernatant was transferred to a tube, and 5 mL Coomassie Brilliant Blue G-250 was added. The reaction mixture was incubated for 30min at 60℃. The absorbance of mixture was measured at 595nm. The soluble protein content was calculated according to the method described by Zhang [64].

## Determination of antioxidant enzyme activity

Fresh plant samples (0.5g) were homogenized in 5 mL Extraction buffer (0.05M Tris-HCl buffer containing 1 mmol/L EDTA, 1 mmol/L dithiothreitol, 1 mmol/L ascorbic acid, 1 mmol/L glutathione, and 5 mmol/L MgCl_2_, 20% glycerinum, pH 7.0) and then centrifuged at 10,000 × g for 10min. The POD, SOD and CAT activities were determined according to manufacturer’s instructions of Assay Kit (Solarbio, Beijing, China).

### Data analyses

All experiments in this study were conducted in at least three replicates. The data were processed with SPSS software version 23.0 (Armonk, NY, USA). The significant differences among experimental groups were analyzed by Student’s t test with P ≤ 0.05.

## Electronic supplementary material

Below is the link to the electronic supplementary material.

Supplementary Material 1

## Data Availability

The datasets generated during the current study are available in the GenBank at NCBI (https://www.ncbi.nlm.nih.gov/), accession number: OM903864-OM903874, OM903083-OM903085, OM908921-OM908926, OM909061-OM909062, OM908937, OM903884, OM909072.

## Abbreviations

**PGPR**: Plant growth promoting rhizobacteria
**JA**: Jasmonate
**SA**: Salicylic acid
**ACC**: 1-aminocyclopropane-1-carboxylate
**IAA**: Indole-3-acetic acid
**MDA**: Malondialdehyde
**SOD**: Superoxide dismutase
**CAT**: Catalase
**POD**: Peroxidase
