# Supplementary material for: The bacterial consortia promote plant growth and secondary metabolite accumulation in Astragalus mongholicus under drought stress
Source: BMC Plant Biol. 2022 Oct 7;22:475. doi: 10.1186/s12870-022-03859-4 (PMC9541091; doi:10.1186/s12870-022-03859-4)
Supplement: Supplementary file 1 — Supplementary Material 1 [file 12870_2022_3859_MOESM1_ESM.docx]

Fig. S1 Proportion of bacterial strains isolated from rhizosphere or root of Astragalus. After 16S sequence alignment of 429 bacterial strains, the proportion of dominant bacteria
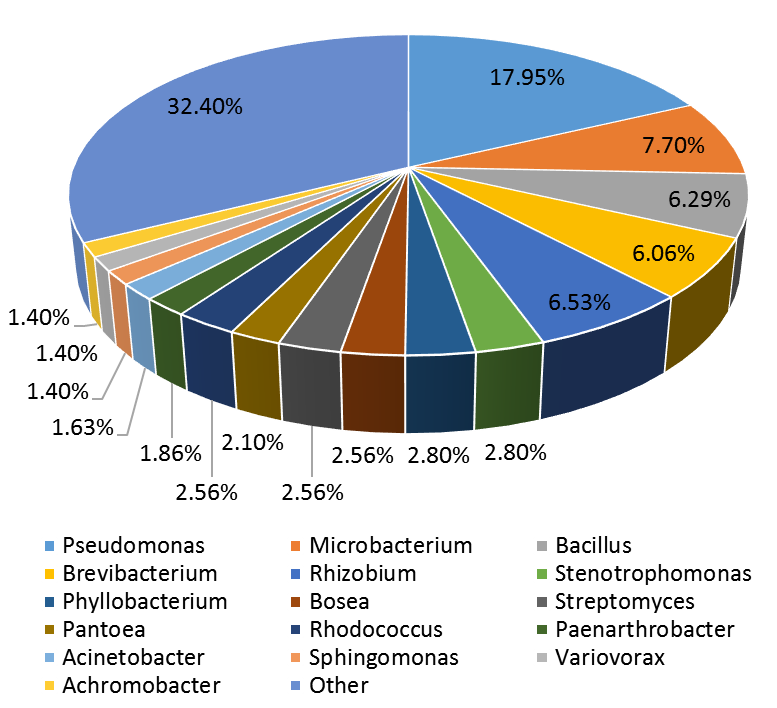
l genera was shown.


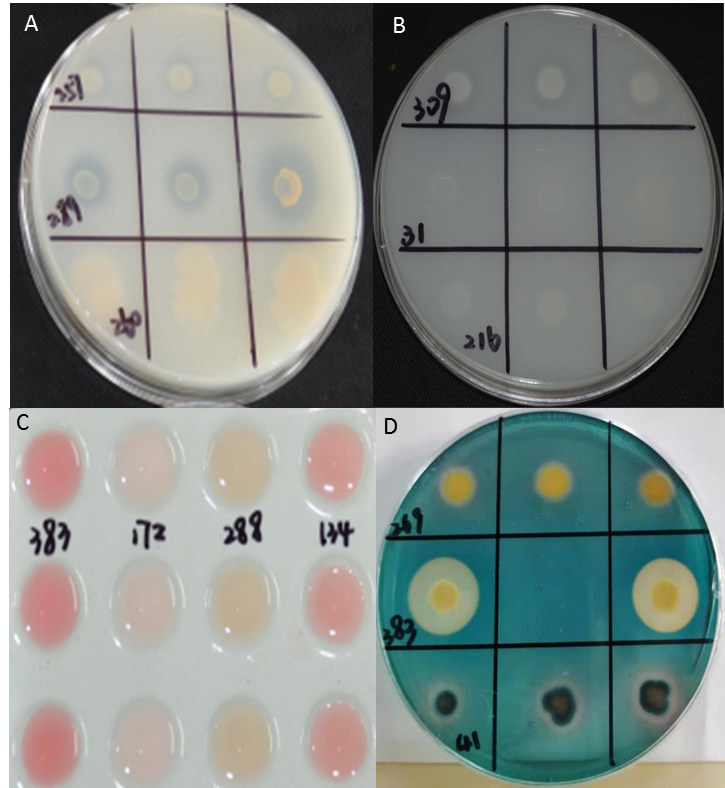
Fig. S2 Preliminary screening of plant growth promoting characteristics of bacterial isolates. A: The phosphate dissolving clear zone around the bacterial colonies. B: The potassium decomposing clear zone around the bacterial colonies. C: Color reaction for IAA in bacterial solution. D: Clear zone around colonies after chelation of iron and siderophores. For phosphate, potassium and siderophores, the ratio of D (Clear zone diameter) / d (Colony diameter) represents the intensity of corresponding function. For IAA, pink means the strain can produce IAA.


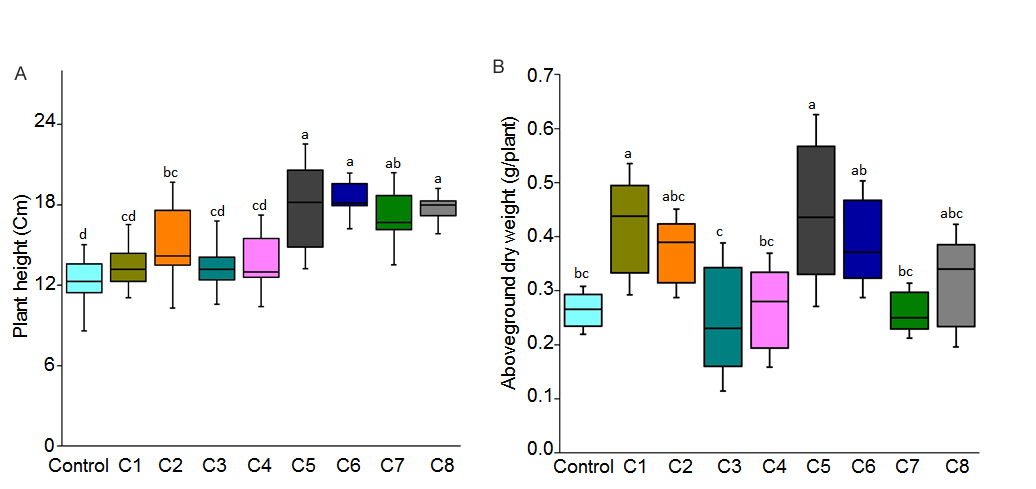
Fig. S3 Effect of different bacterial consortia inoculation on plant height (A) and shoot dry weight (B) of plants inoculated with different bacterial consortia and all plants grew for 5 months. Letters indicate significant differences (Root length, n = 24; Root dry weight, n = 4, P < 0.05). C1-C8, bacterial consortium 1- bacterial consortium 8.


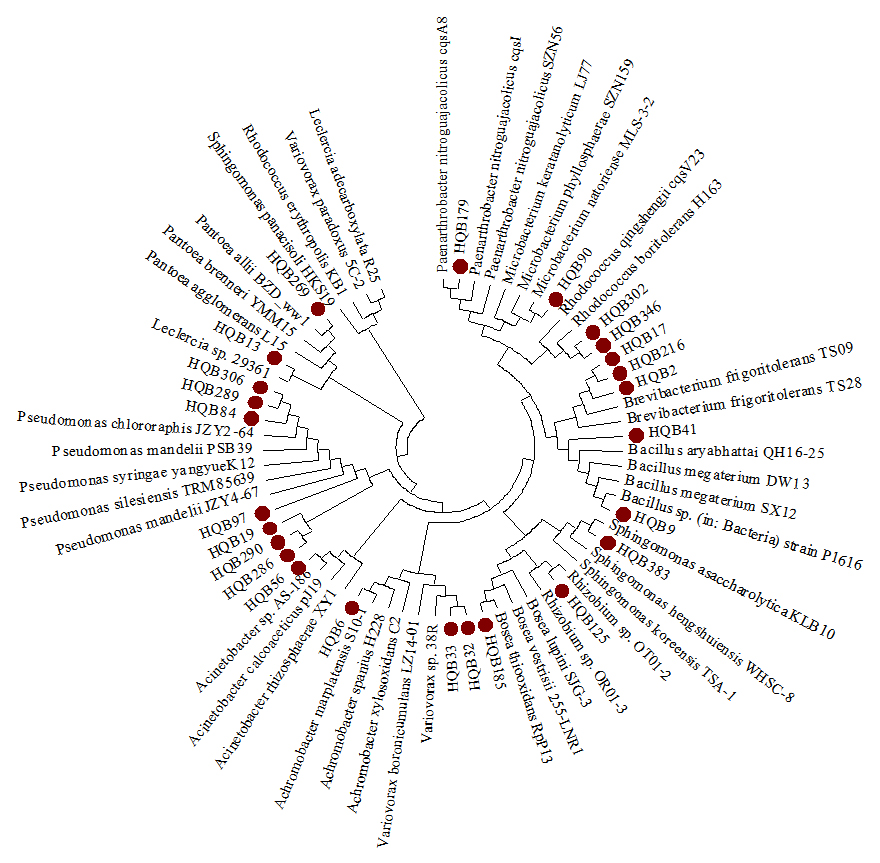
Fig. S4 Bacteria used for consortia construction. Bacterial strains were isolated from Astragalus rhizosphere or roots. Neighbour-joining tree generated according to the 16S rRNA gene sequences of 25 bacterial strains. Red circles indicated the strains were used in our study.


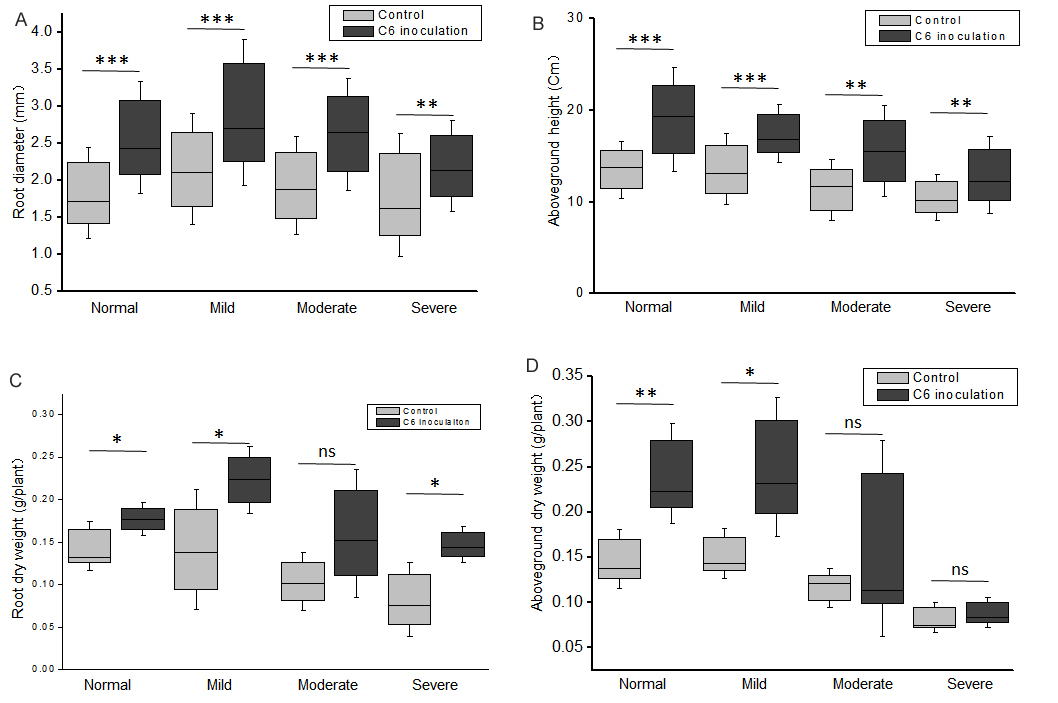
Fig. S5 Effect of drought stress and consortium 6 on the plant growth. Bacterial consortium 6 inoculated or non-inoculated plants grew in soil with different moisture levels. Root diameter (A), root dry weight (C), aboveground height (B) and aboveground dry weight (D) were determined after the plants grew 30 days in drought conditions. ‘ns’ indicated no significant difference. The asterisks indicated significant difference according to Student's t test, P ≤ 0.05; ‘**’: P ≤ 0.01; ‘***’: P ≤ 0.001.


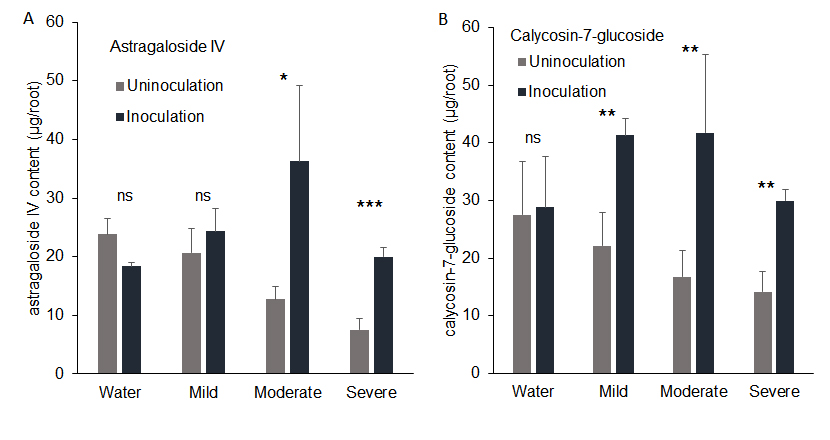
Fig S6 Effect of drought stress and consortium 6 on the total amount of calycosin-7-glucoside and astragaloside IV. Plants grew in soil for two months and subjected to different drought levels for 30 days, the accumulation of total astragaloside IV (A) and calycosin-7-glucoside (B) in un-inoculated and inoculated Astragalus root was determined. Each treatment had four replications. ‘ns’ indicated no significant difference. The asterisks indicated significant difference according to Student's t test, ‘*’: P ≤ 0.05; ‘**’: P ≤ 0.01.

Table S1 Strains contained in the different bacterial consortia

| Isolates | Genus | Consortium 1 | Consortium 2 | Consortium 3 | Consortium 4 | Consortium 5 | Consortium 6 | Consortium 7 | Consortium 8 |
| --- | --- | --- | --- | --- | --- | --- | --- | --- | --- |
| HQB9 | *Bacillus* sp. |  |  |  | + | + | + | + | + |
| HQB56 | *Acinetobacter* sp. |  |  |  |  |  | + | + | + |
| HQB32 | *Variovorax* sp*.* |  |  |  |  |  |  | + | + |
| HQB13 | *Leclercia* sp. |  |  |  | + | + | + | + | + |
| HQB383 | *Sphingomonas* sp. | + | + | + | + | + | + | + | + |
| HQB185 | *Bosea* sp. |  |  |  |  |  |  | + | + |
| HQB41 | *Bacillus* sp. |  |  |  |  |  |  | + | + |
| HQB125 | *Rhizobium* sp. |  |  |  |  |  |  |  | + |
| HQB269 | *Pantoea* sp. |  |  |  |  |  |  |  | + |
| HQB302 | *Rhodococcus* sp. |  |  |  |  |  | + | + | + |
| HQB289 | *Pseudomonas* sp*.* |  | + | + | + | + | + | + | + |
| HQB17 | *Bacillus* sp. |  |  |  |  |  |  |  | + |
| HQB179 | *Paenarthrobacter* sp. |  |  |  |  |  |  |  | + |
| HQB19 | *Pseudomonas* sp*.* | + | + | + | + | + | + | + | + |
| HQB306 | *Pseudomonas* sp*.* |  |  | + | + | + | + | + | + |
| HQB90 | *Microbacterium* sp. |  |  |  |  |  | + | + | + |
| HQB33 | *Variovorax* sp*.* |  |  |  |  |  |  | + | + |
| HQB6 | *Achromobacter* sp. | + |  |  |  |  |  | + | + |
| HQB2 | *Brevibacterium* sp. |  |  |  |  |  |  |  | + |
| HQB290 | *Pseudomonas* sp. |  |  | + | + | + | + | + | + |
| HQB216 | *Brevibacterium* sp. |  | + | + | + | + | + | + | + |
| HQB97 | *Pseudomonas* sp. | + | + | + | + | + | + | + | + |
| HQB84 | *Pseudomonas* sp. |  | + | + | + | + | + | + | + |
| HQB286 | *Pseudomonas* sp*.* |  |  |  |  | + | + | + | + |
| HQB346 | *Rhodococcus* sp. |  |  |  |  | + | + | + | + |

Note：”+” means the consortium contains this strain.

Table S2 Effect on bacterial consortia inoculation on plant growth

|  | Root length (Cm) | | Plant height (Cm) | | Root fresh weight (g) | | Root dry weight (g) | | Aboveground fresh weigh (g) | | Aboveground dry weigh (g) | |
| --- | --- | --- | --- | --- | --- | --- | --- | --- | --- | --- | --- | --- |
|  | F | *P* | F | *P* | F | *P* | F | *P* | F | *P* | F | *P* |
| Consortium 1vs Control | 9.89 | **0.015^*^** | 2.06 | **0.023^*^** | 1.32 | **0.020^*^** | 9.57 | **0.029^*^** | 4.00 | 0.776 | 1.96 | **0.042^*^** |
| Consortium 2vs Control | 1.89 | **0.001^**^** | 0.01 | 0.077 | 0.28 | 0.069 | 9.73 | 0.050 | 0.04 | 0.339 | 3.34 | 0.830 |
| Consortium 3vs Control | 3.34 | **<0.001^***^** | 0.10 | 0.071 | 2.48 | 0.351 | 13.23 | 0.503 | 1.57 | 0.501 | 2.29 | 0.994 |
| Consortium 4vs Control | 9.38 | **0.003^**^** | 1.88 | **<0.001^***^** | 0.03 | **0.013^*^** | 1.39 | **0.007^**^** | 1.06 | **0.037^*^** | 2.94 | 0.059 |
| Consortium 5vs Control | 5.41 | **0.003^**^** | 1.30 | **<0.001^***^** | 3.40 | **0.004^**^** | 3.59 | **0.007^**^** | 1.30 | **0.026^*^** | 3.09 | **0.043^*^** |
| Consortium 6vs Control | 4.32 | **0.002^**^** | 0.02 | **<0.001^***^** | 2.11 | **0.038^*^** | 9.99 | **0.014^*^** | 1.01 | 0.810 | 0.19 | 0.971 |
| Consortium 7vs Control | 3.36 | **<0.001^***^** | 2.76 | **<0.001^***^** | 0.52 | 0.317 | 11.47 | 0.996 | 2.21 | 0.257 | 4.03 | 0.386 |
| Consortium 8vs Control | 2.09 | **0.001^**^** | 0.18 | **0.049^*^** | 0.01 | **0.044^*^** | 5.58 | **0.004^**^** | 1.83 | 0.144 | 3.47 | **0.039^*^** |

Note: Plant grew for 5 months. Root length and plant height n=24; Root dry weight, fresh weight, aboveground fresh weight and aboveground dry

weight n=4; *, means P <0.05，*, means P <0.01，***, means P <0.001.
